# Bushen Zhuangjin Decoction promotes chondrocyte proliferation by stimulating cell cycle progression

**DOI:** 10.3892/etm.2015.2214

**Published:** 2015-01-23

**Authors:** XIHAI LI, JIASHOU CHEN, WENNA LIANG, HUITING LI, FAYUAN LIU, XIAPING WENG, PINGDONG LIN, WENLIE CHEN, CHUNSONG ZHENG, HUIFENG XU, XIANXIANG LIU, HONGZHI YE

**Affiliations:** 1 Academy of Integrative Medicine, Fujian University of Traditional Chinese Medicine, Fuzhou, Fujian 350122, P.R. China; 2 Research Base of Traditional Chinese Medicine Syndrome, Fujian University of Traditional Chinese Medicine, Fuzhou, Fujian 350122, P.R. China; 3 Fujian Key Laboratory of Integrative Medicine on Geriatrics, Fujian University of Traditional Chinese Medicine, Fuzhou, Fujian 350122, P.R. China

**Keywords:** chondrocyte, cell cycle, osteoarthritis, Bushen Zhuangjin Decoction

## Abstract

Bushen Zhuangjin Decoction (BZD), a well-known formulation in Traditional Chinese Medicine, has been widely used for the treatment of osteoarthritis (OA). Due to the poor intrinsic repair capacity of chondrocytes, promoting the proliferation of chondrocytes is an efficient treatment to delay the progression of cartilage degradation. The present study, therefore, focused on the effect of BZD on chondrocyte proliferation, exploring the mechanism of BZD on the inhibition of cartilage degradation. Chondrocytes isolated from the knee articular cartilage of Sprague Dawley rats were cultured and identified by type II collagen immunohistochemistry. It was found that BZD promoted chondrocyte viability in a dose- and time-dependent manner. To investigate if BZD promoted the chondrocyte viability by stimulating the cell cycle progression a flow cytometer was used, and the results showed that the percentage proportion of G0/G1 cells was significantly lower, and the percentage proportion of S cells was significantly higher, in treated cells compared with that in untreated cells. To gain insight into the mechanism underlying the effect of BZD on the cell cycle progression, the mRNA and protein expression of cyclin D1, cyclin-dependent kinase 4 (CDK4), CDK6 and p21 was measured by reverse transcription-polymerase chain reaction and western blotting, respectively. The mRNA and protein expression of cyclin D1, CDK4 and CDK6 in the BZD-treated chondrocytes was significantly upregulated, while the mRNA and protein expression of p21 was significantly downregulated, compared with that in the untreated chondrocytes. These results suggested that BZD promoted chondrocyte proliferation by accelerating G1/S transition, indicating that BZD is a potential therapeutic agent for the treatment of OA.

## Introduction

Osteoarthritis (OA), a common chronic disease increasingly prevalent with age, is manifested by joint pain, stiffness, disability and/or swelling, and reduces the overall quality of life of affected individuals (1,2). Cartilage degradation, an important feature of OA, is caused by the interplay of metabolic, genetic, biochemical and biomechanical factors. This degradation results in the imbalance of catabolism and anabolism, which degrades the structural and functional integrity of the extracellular matrix (ECM). The integrity of the ECM is regulated by chondrocytes, the only type of cell in cartilage (3). Due to the poor intrinsic repair capacity of chondrocytes, articular cartilage damage is often progressive and is generally permanent; therefore, improving and maintaining the proliferation potential and phenotype of chondrocytes may potentially be an effective method to delay the development and progression of OA.

Efficient cell cycle regulation is vital for cell growth and differentiation. The family of cyclin-dependent kinases (CDKs) is one of the key regulators in the cell cycle process (4,5). CDKs pair with cell cycle-specific regulatory subunits known as cyclins to govern the cell cycle. The activity of the CDKs is controlled at multiple levels: Transcriptionally, post-translationally and by CDK inhibitors (CDKIs) (6). Multiple cell cycle genes have been implicated in chondrocyte proliferation, including cyclin D1, CDK4, CDK6 and p21, affecting G1 phase progression as well as S phase entry (7).

Since there are numerous adverse side effects of conventional western medication in the treatment of OA (8), the use of complementary and alternative medicine to improve the signs and symptoms of diseased joints has been widely accepted by patients with OA (9). Traditional Chinese Medicine (TCM) has been used for the treatment of OA in China for thousands of years. According to the theory of TCM, OA is believed to originate in the Jin-Gu (tendons and bones) but presents as Ben-Xu (visceral insufficiency) and Biao-Shi (asthenia in superficiality). The invasion of pathogens due to ‘wind-cold-dampness’ (three pathogenic factors of TCM) is linked to blood stasis, which then leads to Bi Zheng (arythromyodonia) and Wei Zheng (wilting syndrome) (10,11). Bi Zheng is treated by blood activation and wind-cold-dampness purging, and Wei Zheng is treated by visceral reinforcement.

Bushen Zhuangjin decoction (BZD), a TCM formulation, consists of 10 component herbs: 12 g Shu Di Huang (steamed Chinese foxglove, *Radix Rehmanniae Glutinosae Conquitae*), 12 g Dang Gui (*Angelica sinensis*), 10 g Niu Xi (*Achyranthes bidentatae*), 12 g Shan Zhu Yu (*Cornus officinalis*), 12 g Fu Ling (*Poria cocos*), 12 g Xu Duan (*Dipsacus*), 10 g Du Zhong (*Eucommia* bark) 10 g Bai Shao (white peony root, *Radix Paeoniae Alba*), 5 g Qing Pi (immature tangerine peel, *Pericarpium Citri Reticulatae Viride*) and 10 g Wu Jia Pi (*Acanthopanax* root bark). These natural products together confer BZD properties of nourishing Gan (liver) and Shen (kidney) and strengthening tendons and bones, according to the theories of TCM (12). BZD has a long history of use in the treatment of OA, and has been shown to improve osteoarthritic symptoms in clinical trials (12–14); however, the molecular mechanism of BZD in the treatment of OA is complicated and multifaceted and is thus not fully understood. In the present study, the effect of BZD on chondrocyte proliferation was investigated to elucidate the mechanisms underlying the therapeutic effects of BZD in OA.

## Materials and methods

### Animals

Four-week-old male Sprague Dawley rats of Specific Pathogen Free status were purchased from Shanghai SLAC Laboratory Animal Co. (Shanghai, China). The present study was approved by the Institutional Animal Care and Use Committee of Fujian University of TCM (Fuzhou, China).

### Preparation of BZD

The herbs of BZD, obtained from the Third People’s Hospital of Fujian University of TCM (Fuzhou, China), were dried in an air-circulating oven (model SFG-02.600; Hengfeng Medical Instrument Co., Ltd., Huangshi, China) at 50°C for 24 h, and then crushed to an appropriate particle size in a high-speed rotary cutting mill (model ZN-400A; Zhongnan Pharmaceutical Machinery Factory, Changsha, China). According to the proportion of BZD, 105 g herbal powder was extracted with 1,050 ml 85% ethanol using a refluxing method and filtered. The filtrate was evaporated on a rotary evaporator (model RE-2000; Shanghai Yarong Biochemistry Instrument Factory, Shanghai, China) and then dried to a constant weight in a vacuum drying oven (model DZF-300; Shanghai Yiheng Scientific Instrument Co., Ltd., Shanghai, China). The BZD was dissolved in dimethylsulfoxide (DMSO; Hengxing Chemical Preparation Co., Ltd., Tianjin, China) to a stock concentration of 100 mg/ml and stored at −20°C. The working concentrations of BZD were made by diluting the stock solution in Dulbecco’s modified Eagle’s medium (DMEM) containing 10% fetal bovine serum (FBS) (both HyClone Laboratories, Inc., Logan, UT, USA), and then filtering through a 0.22-μm filter and storing at 4°C. The final concentration of DMSO in the DMEM was <0.5%.

### Isolation, culture and identification of chondrocytes

Chondrocytes from the knee articular cartilage of rats were isolated, cultured and identified as previously described (4). Briefly, the chondrocytes from the knee articular cartilage of four-week-old rats were isolated using 0.2% type II collagenase (Sigma-Aldrich, St. Louis, MO, USA) in magnesium- and calcium-free phosphate buffered saline (pH 7.4) (HyClone Laboratories, Inc.) for 1 h at 37°C. The cells were then resuspended in DMEM supplemented with 10% FBS, 100 μg/ml streptomycin and 100 U/ml penicillin (HyClone Laboratories, Inc.), and seeded in a monolayer at a density of 5×10^5^ cells/cm^2^. The second passage cells were identified by type II collagen immunohistochemistry. Briefly, sterilized coverslips were placed into the wells of a 24-well plate, the cells were seeded onto the coverslips (2×10^5^ cells/well) and were cultured for 48 h at 37°C in an incubator containing 5% CO_2_. The cells were then fixed with 4% paraformaldehyde (Sigma-Aldrich) at 4°C for 30 min, and blocked with 10% bovine serum albumin (Sigma-Aldrich) for 30 min. The slides were then incubated with a polyclonal rabbit antibody targeting type II collagen (1:100; BS1071, Bioworld Technology, Inc., St. Louis Park, MN, USA) overnight at 4°C, followed by an incubation with biotinylated goat anti-rabbit secondary antibody immunoglobulin (Ig)G (1:2,000; ZB-2301, Beijing Zhongshan Golden Bridge Biotechnology Co., Ltd., Beijing, China) for 30 min. The slides were then incubated with streptavidin-horseradish peroxidase (HRP) conjugate (Zhongshan Golden Bridge Biotechnology Co., Ltd.) for 30 min, before the antibody-HRP complex was visualized by incubation with diaminobenzidine (ZLI-9018 DAB kit; Zhongshan Golden Bridge Biotechnology Co., Ltd.) for 5 min. The slides were briefly counter-stained with hematoxylin (Sigma-Aldrich) and dehydrated, and cover slips were added. Images were captured under a light microscope (BH2; Olympus, Tokyo, Japan). Chondrocytes were treated with 0, 100, 200 and 400 μg/ml BZD for 48 h.

### Cell viability analysis

The chondrocytes were treated with BZD at different concentrations (0, 50, 100, 200, 300, 400, 600, 800 and 1,200 μg/ml) and for different lengths of time (24, 48 or 72 h). A total of 100 μl 0.5% MTT (Sigma-Aldrich) was added, and the cells were incubated at 37°C for 4 h. The purple-blue MTT formazan precipitate was dissolved in 150 μl DMSO and agitated for 10 min. The absorbance was measured at 490 nm using an ELISA reader (model EXL800, BioTek, Winooski, VT, USA).

### Cell cycle analysis

The cell cycle of the chondrocytes was determined with a cell cycle assay kit (KeyGEN Biotech, Nanjing, China) using a fluorescence-activated cell sorting (FACS) machine (FACSCaliber™; Becton-Dickinson, San Diego, CA, USA). Staining was performed according to the manufacturer’s instructions. The percentage of cells in the different phases was calculated using ModFit software (Verity Software House, Topsham, ME, USA) including the G0/G1, S, G2 and M phases.

### RNA extraction and reverse transcription-polymerase chain reaction (RT-PCR) analysis

The total RNA was extracted with TRIzol™ reagent (Invitrogen Life Technologies, Carlsbad, CA, USA) and reverse transcribed into complementary DNA with SuperScript^®^ II reverse transcriptase (Promega Corp, Madison, WI, USA). The analysis of the mRNA expression of cyclin D1, CDK4, CDK6 and p21 was performed using PCR. The primers (Sangon Biotech Co., Ltd., Shanghai, China) used for the PCR were as follows: Cyclin D1 forward, 5′-AAT GCC AGA GGC GGA TGA GA-3′ and reverse, 5′-GCT TGT GCG GTA GCA GGA GA-3′ (189 bp); CDK4 forward, 5′-GAA GAC GAC TGG CCT CGA GA-3′ and reverse, 5′-ACT GCG CTC CAG ATT CCT CC-3′ (109 bp); CDK6 forward, 5′-TTG TGA CAG ACA TCG ACG AG-3′, and reverse, 5′-GAC AGG TGA GAA TGC AGG TT-3′ (151 bp); p21 forward, 5′-CGG GCA GTC CCT TCT AGT TCC-3′ and reverse, 5′-AAT GCT TGA GCA CAC ACG AG-3′ (204 bp); β-actin forward, 5′-CGT TGA CAT CCG TAA AGA CC-3′ and reverse, 5′-GGA GCC AGG GCA GTA ATC T-3′ (108 bp). The DNA bands were examined using a Gel Documentation system (Model Gel Doc 2000; Bio-Rad, Hercules, CA, USA) and normalized to β-actin.

### Western blot analysis

Total proteins were collected immediately in lysis buffer (Beyotime Institute of Biotechnology, Haimen, China), stored for 30 min on ice and quantified using the bicinchoninic acid assay. A total of 20 μg protein was separated on a 12% SDS-PAGE gel and transferred onto polyvinylidene fluoride membranes. The membranes were blocked, and incubated with rabbit polyclonal antibodies targeting cyclin D1 (1:1,000; sc-718), CDK4 (1:1,000; sc-260), CDK6 (1:1,000; sc-177), p21 (1:1,000; sc-397) and β-actin (1:1,000; sc-7210) (Santa Cruz Biotechnology, Inc., Santa Cruz, CA, USA) overnight at 4°C. Goat anti-rabbit horseradish peroxidase-conjugated secondary antibody IgG (1:10,000; ZB-2301, Zhongshan Golden Bridge Biotechnology Co., Ltd.) was added to the membranes at room temperature. The immunocomplexes were visualized by the enhanced chemiluminescence method. The bands were quantified by scanning densitometry (Molecular Imager ChemiDoc X-Ray Spectroscopy System, cat. no. 170-8070; Bio-Rad). Protein concentrations were determined using the Tocan 190 protein assay system (Bio-Rad) and normalized to β-actin.

### Statistical analysis

All data are presented as the mean ± standard deviation from at least three independent experiments. Statistical analysis was performed using SPSS software (version 13.0; SPSS Inc., Chicago, IL, USA) with one-way analysis of variance, and multiple comparisons were performed with the Student-Newman-Keuls q test. P<0.05 was considered to indicate a statistically significant difference.

## Results

### Morphology and identification of the chondrocytes

**Figure 1:**
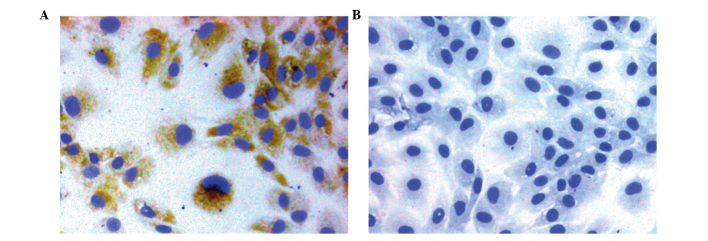
Chondrocyte identification by type II collagen immunohistochemistry. (A) Positive chondrocytes stained brown in the cytoplasm; (B) chondrocytes negative for type II collagen did not stain. Magnification, ×200.

The chondrocytes exhibited a typical morphology with a polygonal or spherical shape, as described in previous studies (4,15). The type II collagen in the cytoplasm of the passage 2 chondrocytes was stained brown, which represented positive expression; no staining was observed in the negative control cells (Fig. 1A and B).

### BZD enhances chondrocyte viability and promotes cell cycle progression

**Figure 2:**
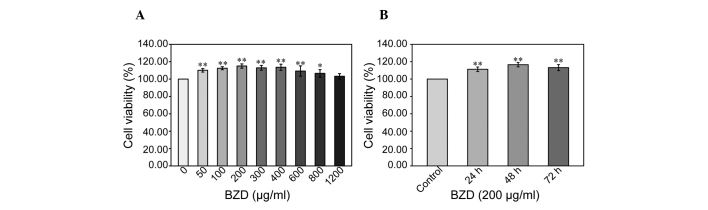
BZD enhances the cell viability of chondrocytes. (A) Chondrocytes were treated with different concentrations of BZD for 48 h. (B) Chondrocytes were treated with 200 μg/ml BZD for the indicated time periods. Data are presented as the mean ± standard deviation, shown as vertical bars. ^*^P<0.05 and ^**^P<0.01, compared with untreated cells. BZD, Bushen Zhuangjin Decoction.

To explore the effect of BZD on chondrocyte viability, the cell viability of chondrocytes treated with BZD at different concentrations and for different lengths of time was examined using an MTT assay. The cells were treated with BZD concentrations of 50 μg/ml (cell viability, 110.10±2.07%), 100 μg/ml (cell viability, 112.51±1.76%), 200 μg/ml (cell viability, 115.03±2.45%), 300 μg/ml (cell viability, 112.91±2.76%), 400 μg/ml (cell viability, 113.60±3.53%), 600 μg/ml (cell viability, 109.25±5.96%), 800 μg/ml (cell viability, 106.55±4.32%) and 1,200 μg/ml (cell viability, 103.25±3.03%) for 48 h. A dose-dependent increase in cell viability was observed following BZD treatment compared with untreated cells (100±0.00%) (P<0.05 or P<0.01) (Fig. 2A). Cell viability was gradually enhanced in chondrocytes treated with 200 μg/ml BZD for 24, 48 and 72 h (Fig. 2B), indicating that BZD promotes chondrocyte viability in a dose- and time-dependent manner.

**Figure 3:**
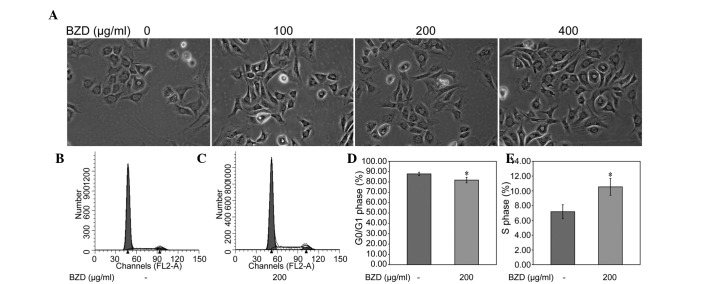
BZD promotes cell cycle progression. (A) Morphological changes of chondrocytes treated with different concentrations of BZD for 48 h were observed under a phase-contrast microscope (magnification, ×200). (B and C) Chondrocytes treated with or without BZD were stained with propidium iodide followed by fluorescence-activated cell sorting analysis. The percentage of (D) G0/G1- and (E) S-phase cells in the chondrocytes treated with or without BZD. Data are presented as the mean ± standard deviation, shown as vertical bars. ^*^P<0.05, compared with the untreated cells. BZD, Bushen Zhuangjin Decoction.

The morphology of the treated chondrocytes was observed by inverted microscopy. The results showed that the treated chondrocytes underwent morphological changes, including in cell shape and size, compared with the untreated chondrocytes. A few cells were additionally observed to contain two nuclei (Fig. 3). To investigate whether BZD promoted the chondrocyte viability by stimulating cell cycle progression, the cell cycle of the chondrocytes treated with BZD was examined using FACS analysis. The percentage proportion of G0/G1 cells was significantly lower, and the percentage proportion of S cells was significantly higher, in treated cells compared with that in untreated cells (P<0.05) (Fig. 3B–E), indicating that BZD promotes chondrocyte viability by stimulating cell cycle progression.

### BZD upregulates the expression of cyclin D1, CDK4 and CDK6 and downregulates the expression of p21

**Figure 4:**
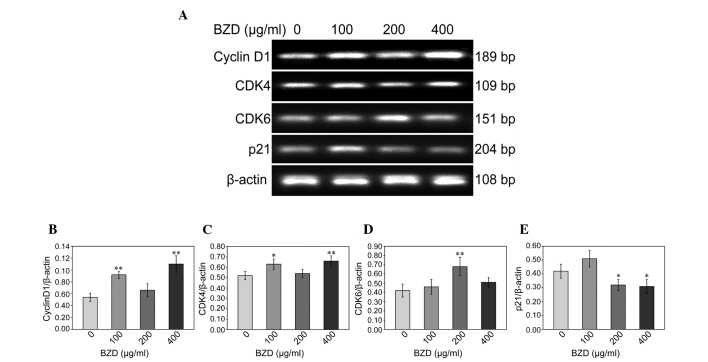
BZD affects the mRNA expression of cell cycle regulators in chondrocytes. (A) Reverse transcription-polymerase chain reaction showing the mRNA expression of cell cycle regulators in chondrocytes treated with or without BZD. (B-E) mRNA expression of (B) cyclin D1, (C) CDK4, (D) CDK6 and (E) p21 in chondrocytes treated with or without BZD. β-actin was used as the reference mRNA for the quantification analysis. Data are presented as the mean ± standard deviation, shown as vertical bars. ^*^P<0.05 and ^**^P<0.01, compared with untreated cells. BZD, Bushen Zhuangjin Decoction; CDK, cyclin-dependent kinase.

**Figure 5:**
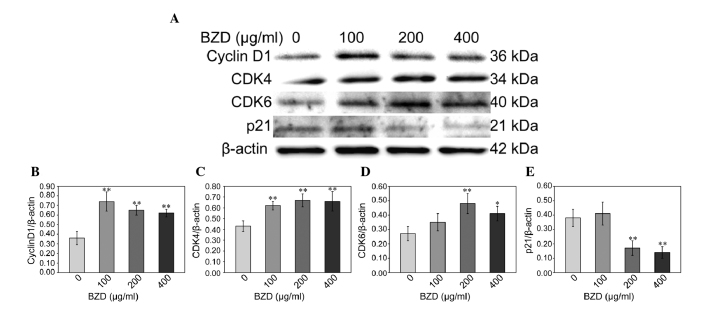
BZD affects the protein levels of the cell cycle regulators in chondrocytes. (A) Western blot analysis showing the protein expression of cell cycle regulators in chondrocytes treated with or without BZD. (B-E) Protein levels of (B) cyclin D1, (C) CDK4, (D) CDK6 and (E) p21 in chondrocytes treated with or without BZD. β-actin was used as the reference protein for the quantification analysis. Data are presented as the mean ± standard deviation, shown as vertical bars. ^*^P<0.05 and ^**^P<0.01, compared with untreated cells. BZD, Bushen Zhuangjin Decoction; CDK, cyclin-dependent kinase.

To gain an insight into the effect of BZD on the cell cycle of chondrocytes, the mRNA and protein expression of cyclin D1, CDK4, CDK6 and p21 was analyzed using RT-PCR and western blotting. Compared with the untreated cells, the mRNA expression of cyclin D1, CDK4 and CDK6 in the BZD-treated chondrocytes was significantly upregulated (P<0.01 or P<0.05), while the mRNA expression of p21 was significantly downregulated (P<0.05) (Fig. 4). The protein levels of cyclin D1, CDK4, CDK6 and p21 were similar to their respective mRNA expression (Fig. 5).

## Discussion

OA, belonging to the category of Gu Bi (bone impediment) in TCM, is based on a deficiency of the liver and kidney (16,17). BZD can nourish the kidney, soothe the liver, promote blood circulation and dispel wind, and acts against the pathogenesis of OA, with kidneys dominating bones and the liver governing tendons, according to the theory. It was found in the present study that BZD promoted chondrocyte viability in a dose- and time-dependent manner by stimulating cell cycle progression, indicating that BZD is a potential therapeutic agent for the treatment of OA.

Alternations in gene and protein expression in articular chondrocytes are likely to play an important role in the pathological process of OA. Cartilage has minimal reparative capacity, and the degradation of articular cartilage can have severe consequences. Chondrocytes produce and maintain the ECM, which is responsible for providing the appropriate function to articular cartilage (18,19). For this reason, the differentiation, proliferation and apoptosis of chondrocytes are crucial to the maintenance of the cartilage function, and the functional changes of chondrocytes play important roles contributing to the degeneration of the joint cartilage. Previous studies have focused on the promotion of chondrocyte proliferation as an efficient treatment to delay the progression of cartilage degradation (4,15); thus, the effect of BZD on the proliferation of chondrocytes was investigated in the present study.

The MTT data showed that BZD promoted chondrocyte viability in a dose- and time-dependent manner; flow cytometry detected changes in the cell cycle with a greater sensitivity than MTT. The results showed that the percentage of chondrocytes in the G0/G1 phase was significantly decreased, and the percentage of chondrocytes in the S phase was significantly increased, in treated versus untreated cells. This indicates that BZD promotes the cell cycle progression of chondrocytes *in vitro*, thus enhancing chondrocyte proliferation.

The cell cycle refers to the series of events that take place in a cell that leads to its division and duplication (replication), and results in the production of two daughter cells (20). Cell cycle regulation can be divided into exogenous and endogenous regulation. The former is primarily caused by cytokines and external stimuli, while the latter is mainly realized through a complicated network by cyclin, CDKs and CDKIs (21). There are two restriction points in the cell cycle. One begins at the initial synthesis of DNA, which is the G1/S restriction point; the other starts at the beginning of mitosis, i.e. the G2/S restriction point. The G1/S restriction point is more important since it is key to cell proliferation whether cells can pass through it (22). Cyclin D1 is the positive regulatory factor for the switch of the G1/S phase in the cell cycle and combines with CDK4 to promote the cell cycle process. In addition, p21 competes with cyclin D1 in the combination with CDK4 to prevent Rb from being phosphorylated, leading to a block at the G1 phase. Cyclin D1, CDK4 and p21, therefore, are all key proteins of cell cycle signals at the G1 phase, and the G1/S restriction point determines whether cells will divide or activate the mechanism for apoptosis (23,24). The effect of BZD on the mRNA and protein expression of cyclin D1, CDK4, CDK6 and p21 was also studied and the results showed that BZD enhances cyclin D1, CDK4 and CDK6 expression and reduces p21 expression in chondrocytes.

In conclusion, the present data demonstrated that BZD can promote chondrocyte proliferation by upregulating the expression of the positive regulators cyclin D1, CDK4 and CDK6 and downregulating the expression of the negative regulator p21. Based on these results, future experiments are necessary to focus on investigating the effect of BZD on the chondrocyte function in OA *in vivo*.
